# A new health prediction model for a sensor network based on belief rule base with attribute reliability

**DOI:** 10.1038/s41598-021-82594-6

**Published:** 2021-02-02

**Authors:** Shaohua Li, Jingying Feng, Wei He, Ruihua Qi, He Guo

**Affiliations:** 1 School of Software Technology, Dalian University of Technology, Dalian, 116620 China; 2 School of Innovation and Entrepreneurship, Dalian University of Foreign Languages, Dalian, 116044 China; 3 School of Software, Dalian University of Foreign Languages, Dalian, 116044 China; 4 Police Information Department, Liaoning Police Academy, Dalian, 116036 China; 5 School of Computer Science and Information Engineering, Harbin Normal University, Harbin, 150025 China

**Keywords:** Computer science, Software

## Abstract

Health prediction plays an essential role in improving the reliability of a sensor network by guiding the network maintenance. However, affected by interference factors in the real operational environment, the reliability of the monitoring information about the sensor network tends to decline, which affects the health prediction accuracy. Furthermore, the lack of monitoring information and high complexity of the network increase the difficulty of health prediction. To solve these three problems, this paper proposes a new sensor network health prediction model based on the belief rule base model with attribute reliability (BRB-r). The BRB-r model is an expert system that fully considers the qualitative knowledge and quantitative data of the sensor network. In addition, it can address the fuzziness and nondeterminacy of this qualitative knowledge. In the new model, the unreliable monitoring information of the sensor network is handled by the attribute reliability mechanism. The reliability of the sensor is calculated by the average distance method. Due to the effect of the fuzziness and nondeterminacy of expert knowledge, the health status of the sensor network cannot be accurately estimated by the initial health prediction model. Consequently, the optimization model for the health prediction model is established. Finally, a case study regarding a sensor network for oil storage tanks is conducted, and the validity of this method is demonstrated.

## Introduction

With the rapid improvement of information technology, sensor networks have been widely applied in engineering practice to gather system information for decision making with respect to maintenance. The reliability and stability of a sensor network directly correlate with the safety of the system. Thus, there is a need to improve the reliability and stability of the sensor network^1–4^.

In an actual work environment, health management is a critical method for improving the reliability and stability of the sensor network. Monitoring information is combined to generate a network’s health status or other features^5–8^. In the literature on the health management of a sensor network, many studies have been conducted and can be classified into three categories: observation data-based methods, knowledge-based methods, and semiquantitative information-based methods^9,10^. For example, Jin et al. proposed a new fault diagnosis approach for sensor networks based on the autoregressive model^11^. Mani et al*.* developed a new robust sensor network for fault diagnosis with uncertain probability data^12^. Hu et al*.* designed a new fault diagnosis model for wireless sensor networks with a new optimization model^1^. In these studies, three problems can be found. First, for sensor networks in engineering practice, due to the manufacturing industry's high reliability, the probability of sensor failure is low, and the available fault data are limited^13–15^. Thus, most of the collected observation information of the sensor network is from standard samples that cannot supply sufficient information to accurately construct a health prediction model. Thus, there is a demand for additional information to make health predictions for sensor networks. Second, a sensor network is applied to supervise the running state of a complex system, and the sensors are installed across a wide range. For an existing system, many interference factors affect the health status of a sensor network, and experts cannot provide accurate knowledge for it. Third, in engineering practice, the system information of the sensor network may be affected by the environment, and there may be noise in the observation information. In other words, the observation information cannot accurately represent the system state, and it is not entirely reliable^16^. Therefore, to improve the health prediction accuracy of a sensor network, three problems must be solved: the lack of observation data, the high complexity of the system, and environmental noise.

The belief rule base (BRB) model is developed based on the IF–THEN rule, fuzzy theory, and evidential reasoning (ER) algorithm^17,18^. It is an expert system that can simultaneously combine expert knowledge and monitoring data. The premise of this model is that the inputs are fully reliable. Feng et al. proposed the BRB-r model, which introduced the attribute reliability mechanism into the BRB model^13^, to enhance its applicability. When the attributes are entirely reliable, the BRB model is a particular case of the BRB-r model. The newly proposed attribute reliability mechanism is used to represent the reliability of the observation information regarding system features. Thus, the BRB-r model provides a practical approach to solve these three problems with respect to health prediction for a sensor network. According to the sensor network features, this article proposes a new attribute reliability calculation method based on the average distance of the observation data. Then, a BRB-r based health status prediction model for the sensor network is proposed, where the attribute reliability mechanism considers the environmental disturbance factors present. Finally, to address the effect of the fuzziness and nondeterminacy of expert knowledge, an optimization model is established based on the projection covariance matrix adaption evolution strategy (P-CMA-ES) algorithm^19^. Since the parameters of the BRB-r model have special physical meaning, the parameters in the health status prediction model should be optimized under certain constraints^20–22^. There are differences and correlations between fuzziness and nondeterminacy. In the sensor network health prediction, fuzziness denotes the uncertainty of experts about the extent of an event itself. For example, the health state of the network may be too large or too small. However, the nondeterminacy represents that certain types of outcomes can occur, but there is uncertainty about which outcome will occur. Meanwhile, fuzziness is also the cognitive nondeterminacy of factors.

The remainder of this article is organized as follows. In "Problem formulation" section, the problems regarding health prediction for sensor networks are formulated. In "Reasoning behind the health prediction model for a sensor network", the reasoning process of the BRB-r model is explained. "Case study" section conducts a case study to demonstrate the potential applications of the developed model. The article is concluded in "Conclusion" section.

## Problem formulation

The health status of a sensor network represents its comprehensive state, and it can be used to assist with decision making regarding system maintenance. In this section, the problems in engineering practice with respect to the sensor network health prediction are described, and a new health prediction model based on BRB-r is proposed.

### Problem formulation regarding health status prediction for sensor networks

In engineering practice, the problems in sensor network health prediction can be formulated as follows.

#### Problem 1

Due to disturbance factors, only a tiny amount of monitoring data is available. Furthermore, the high design reliability and low failure probability of a sensor network result in a minimal amount of failure data, so the network cannot supply sufficient information to accurately construct a health prediction model^1^. Complex disturbance factors that affect a sensor network also make it challenging to accurately build a mathematical model. Therefore, aggregating the gathered observation information and expert knowledge is the first problem to solve.

#### Problem 2

In the process of combining expert knowledge and monitoring data, the fuzziness, nondeterminacy, and incompleteness of expert knowledge and unreliable monitoring data increase the difficulty of health status prediction^13^. Thus, the following health prediction model must be established.

1 $$ H(t + 1) = \Psi (x_{1} (t),x_{2} (t),\ldots,x_{M} (t),r,v) $$

where $H(t + 1)$ is the predicted health status of the sensor network; $\Psi$ is the nonlinear function of the health status prediction model; $x_{1} (t),x_{2} (t),\ldots,x_{M} (t)$ are the features of the sensor network at time instant $t$; $r$ is the expert knowledge applied in the health prediction model; $v$ is the unknown parameters.

#### Problem 3

For complex sensor networks, accurate knowledge can hardly be provided. When uncertain expert knowledge is included in the health prediction model, its prediction accuracy is affected. Therefore, the third problem is how to adjust the health prediction model constructed by experts.

### Health prediction model based on BRB-r for sensor networks

**Figure 1:**
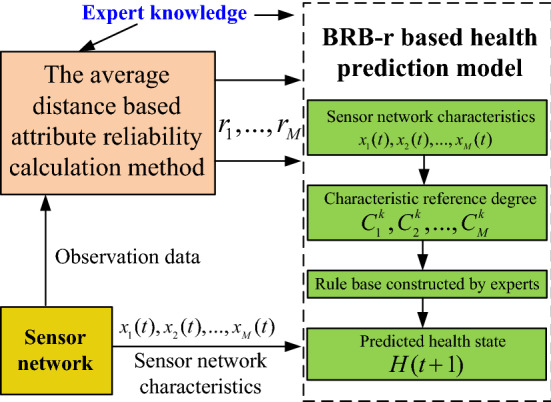
BRB-r based health prediction model for a sensor network.

To address the problems in health prediction for sensor networks, this paper proposes a new sensor network health prediction model based on BRB-r. There are several belief rules in the health prediction model. The $k$th belief rule can be profiled as:

2 $$ \begin{aligned} & R_{k} :{\text{If }}\;x_{1} (t)\;{\text{is }}A_{1}^{k} \wedge x_{2} (t){\text{ is }}A_{2}^{k} \cdots \wedge x_{M} (t){\text{ is }}A_{M}^{k} {, } \\ & \quad \;{\text{Then }}H(t + 1)\;{\text{is }}\left\{ {\left( {D_{1} ,\beta_{1,k} } \right), \ldots ,\left( {D_{N} ,\beta_{N,k} } \right),\left( {D,\beta_{D,k} } \right)} \right\} \\ & \quad \;{\text{With}}\;{\text{rule}}\;{\text{weight}}\;\theta_{k} {,}\;{\text{attribute}}\;{\text{weight }}\delta_{1} , \ldots \delta_{M} ,\;{\text{attribute}}\;{\text{reliability}}\;r_{1} ,\ldots,r_{M} \\ \end{aligned} $$

where $x_{1} (t),x_{2} (t), \ldots ,x_{M} (t)$ are the sensor network features, and $A_{1}^{k} ,A_{2}^{k} , \cdots ,A_{M}^{k}$ are their corresponding reference points. In the sensor network, different features have different situations. Thus, in the BRB-r model, the reference points are used to transform the input information into a uniform framework. $H(t + 1)\;$ represents the predicted health status at time instant $t + 1$. $\left\{ {\left( {D_{1} ,\beta_{1,k} } \right), \ldots ,\left( {D_{N} ,\beta_{N,k} } \right),\left( {D,\beta_{D,k} } \right)} \right\}$ are the output of the $k{\text{th}}$ belief rule. $\left\{ {D_{1} ,D_{2} \ldots ,D_{N} } \right\}$ are the health grades, and $\left\{ {\beta_{1,k} ,\beta_{2,k} , \ldots ,\beta_{N,k} } \right\}$ are the corresponding belief degrees ^23^. The belief degree of the $n{\text{th}}$ health grade represents the likelihood that the sensor network is actually in this grade. $\beta_{D,k}$ is the residual belief degree, and which health grades should be assigned cannot necessarily be determined. In other words, based on the gathered information, the residual belief degree represents the likelihood that the belief rule cannot distinguish the health state of the network ^24–26^. $\sum\nolimits_{n = 1}^{N} {\beta_{n,k} } + \beta_{D,k} = 1$. $\theta_{k}$ is the weight of the belief rule; $\delta_{1} , \ldots \delta_{M}$ are the weights of the input features, and $r_{1} , \ldots ,r_{M}$ are their corresponding reliabilities. The feature weight and feature reliability represent two aspects: subjective and objective aspects. The feature weight can be determined by experts, while the feature reliability is determined by the environment. The structure of the BRB-r based health prediction model is shown in Fig. 1.

#### *Remark 1*

In the developed health prediction model, the attribute weight and reliability denote the subjective and objective aspects of the features of the sensor network, respectively. In engineering practice, the reliabilities of the features are calculated with observation data, while the weights of the features are determined by experts. The difference between these two aspects is explained with an example by Zhou et al.^13^*.*

## Reasoning behind the health prediction model for a sensor network

The reasoning process behind the health prediction model for a sensor network is presented in this section. In "The average distance-based feature reliability calculation method" section, the calculation method for the features of a sensor network is developed. In "BRB-r-based health prediction model" section, the reasoning process behind the health prediction model is described. In "Optimization model for the health prediction model" section, an optimization model for the new health prediction model is constructed. In "Modeling process of the developed health prediction model" section, a complex system modeling method is deduced based on the new model.

### The average distance-based feature reliability calculation method

For a sensor network, its observation data change according to the system state. When the system state changes, the observation data gathered by the sensor network also change. Once the system state is stable at a certain time, the monitoring data of the sensor network should be maintained within specific bounds. Assume that the system is standard. Once the sensor network is affected by environmental interference factors, the gathered monitoring data will fluctuate, and the reliability of these data will decrease. Thus, influenced by the environment, the observation data have noise and cannot accurately reflect the actual system state. There are multiple calculation methods to calculate the reliability of a feature, including the statistics method and expert knowledge method. However, the sensor network is used to monitor the observation data of the system, and the observation data change according to the system state. In current feature reliability calculation methods such as the statistics method, it is assumed that the system state does not change. Thus, these methods cannot calculate the feature reliability of the sensor network. The distances between observation data increase, which demonstrates the reliability of the examined feature^26^. Hence, in this paper, the average distance method is applied to calculate the feature reliability. We assume that the disturbance factors caused by the system environment do not change, and the feature reliabilities are constants.

First, the observation data for the $i$th feature $x_{i} \,$ over T observation points are $x_{i} (1), \ldots ,x_{i} (t), \ldots ,x_{i} (T)$, $i \in \{ 1, \cdots ,M\}$. The distance between the *t*th observation data for the *i*th feature $x_{i} (t)$ and the $t^{\prime}$th observation data point $x_{i} (t^{\prime})$ can be calculated by

3 $$ d_{i} (x_{i} (t),x_{i} (t^{\prime})) = |x_{i} (t) - x_{i} (t^{\prime})| $$

where $t,t^{\prime} \in \{ 1, \ldots ,T\}$, and $T$ are the values in the monitoring data for the *i*th feature.

The average distances among all observation data for the *t*th group observation data of the *i*th feature are obtained by

4 $$ D_{{x_{i} (t)}}^{i} = \frac{1}{T}\sum\limits_{t = 1}^{T} {d_{i} (x_{i} (t),x_{i} (t^{\prime}))} $$

Then, the reliability of the *t*th monitoring data for the *i*th feature is

5 $$ r_{t}^{i} = \frac{{D_{{x_{i} (t)}}^{i} }}{{\max (D_{{x_{i} }}^{i} )}} $$

Using these numerical calculus and derivation, the reliability of the monitoring data for the feature can be calculated. Then, the mean reliability of the *i*th feature can be calculated as follows^26^

6 $$ r_{i} = \frac{1}{T}\sum\limits_{t = 1}^{T} {r_{t}^{i} } ,\;i = 1,2,\ldots,M $$

where $r_{i}$ is the mean reliability of the *i*th feature in a sensor network; $M$ is the total number of sensor network features.

### BRB-r-based health prediction model

The reasoning process behind the health prediction model based on BRB-r is provided, and it is summarized in the following steps in this subsection.

- *Step 1* Calculate the matching degrees between the features and the belief rule. The features of the gathered monitoring data cannot be directly combined because they are in different formats. They should be transformed into the matching degree of the feature reference points by the following equation.
  7 $$ m_{j}^{i} = \left\{ {\begin{array}{*{20}l} {\frac{{A_{i(k + 1)} - x_{i}^{*} (t)}}{{A_{i(k + 1)} - A_{ik} }}} \hfill & \begin{gathered} j = k\quad {\text{if}}\quad A_{ik} \le x_{i}^{*} (t) \le A_{i(k + 1)} \hfill \\ \hfill \\ \end{gathered} \hfill \\ {\begin{array}{*{20}l} {\frac{{x_{i}^{*} (t) - A_{ik} }}{{A_{i(k + 1)} - A_{ik} }}} \hfill \\ \end{array} } \hfill & {j = k + 1} \hfill \\ 0 \hfill & {j = 1,2,\ldots,L,j \ne k,k + 1} \hfill \\ \end{array} } \right. $$
  where $m_{j}^{i}$ is the matching degree between the *i*th feature and the $j{\text{th}}$ rule. $A_{ik}$ and $A_{i(k + 1)}$ are the reference points in the $k{\text{th}}$ and $(k + 1){\text{th}}$ belief rules, respectively. The reference points should be determined by the experts according to the observation data of the features. $x_{i}^{*} (t)$ is the observation data of the *i*th feature, and $L$ is the number of belief rules in the constructed model^27–30^.
  Thus, the matching degree between the *k*th belief rule and the *i*th network feature can be calculated by
  8 $$ \overline{\delta }_{i} = \frac{{\delta_{i} }}{{\mathop {\max }\limits_{{i = 1,\ldots,T_{k} }} \{ \delta_{i} \} }},\quad 0 \le \overline{\delta }_{i} \le 1 $$
  9 $$ C_{i} = \frac{{\overline{\delta }_{i} }}{{1 + \overline{\delta }_{i} - r_{i} }},\;i = 1,2,\ldots,M $$
  10 $$ m_{k} = \prod\limits_{i = 1}^{M} {(m_{k}^{i} )^{{C_{i} }} } $$
  where $m_{k}$ is the matching degree between the $k{\text{th}}$ rule and the *i*th network feature; $\overline{\delta }_{i}$ is the relative weight of the *i*th network feature; $M$ is the number of network features in the health prediction model^13^; $C_{i}$ is the new feature weight considering both feature weight $\overline{\delta }_{i}$ and feature reliability $r_{i}$.
- *Step 2* Calculate the activation weight of the belief rule. Once the matching degrees between the network features and the reference points are obtained, the validity of the belief rules can be expressed by their corresponding activation weights and calculated by
  11 $$ w_{k} = \frac{{\theta_{k} m_{k} }}{{\sum\nolimits_{l = 1}^{L} {\theta_{l} m_{l} } }}\;\;\;\;k = 1,\ldots,L $$
  where $w_{k}$ is the activation weight of the $k{\text{th}}$ rule; $\theta_{k}$ is the rule weight of the $k{\text{th}}$ rule^19,21^.
- *Step 3* Combine the outputs of the belief rules to generate a health status. As shown in Eq. (4), each belief rule has its own output belief degree regarding the predicted health status, which must be combined to produce the final health status of the sensor network. The output belief degrees of the belief rules are combined by the ER algorithm as follows.
  12 $$ \beta_{n} = \frac{{\mu \left[ {\prod\nolimits_{k = 1}^{L} {\left( {w_{k} \beta_{n,k} + 1 - w_{k} \sum\nolimits_{j = 1}^{N} {\beta_{j,k} } } \right)} - \prod\nolimits_{k = 1}^{L} {\left( {1 - w_{k} \sum\nolimits_{j = 1}^{N} {\beta_{j,k} } } \right)} } \right]}}{{1 - \mu \left[ {\prod\nolimits_{k = 1}^{L} {(1 - w_{k} )} } \right]}} $$
  13 $$ \mu = \left[ {\sum\limits_{n = 1}^{N} {\prod\limits_{k = 1}^{L} {\left( {w_{k} \beta_{n,k} + 1 - w_{k} \sum\limits_{j = 1}^{N} {\beta_{j,k} } } \right)} } - (N - 1)\prod\limits_{k = 1}^{L} {\left( {1 - w_{k} \sum\limits_{j = 1}^{N} {\beta_{j,k} } } \right)} } \right]^{ - 1} $$
  where $\beta_{n}$ is the predicted belief degree of the $n{\text{th}}$ health grade $D_{n}$ at time instant t + 1. $\sum\nolimits_{n = 1}^{N} {\beta_{n} \le 1}$^24,25^. When the output of the health prediction model is complete, $\sum\nolimits_{n = 1}^{N} {\beta_{n} = 1}$; otherwise, $\sum\nolimits_{n = 1}^{N} {\beta_{n} < 1}$.
  Then, the predicted health status of the sensor network at time instant $t + 1$ can be calculated as follows
  14 $$ H(t + 1) = \sum\limits_{n = 1}^{N} {u(D_{n} )} \beta_{n} $$
  where $H(t + 1)$ is the predicted health status; $u(D_{n} )$ is the utility of the $n{\text{th}}$ health grade $D_{n}$, and it is determined by the experts according to the decision-making requirements for the subsequent maintenance.

### Optimization model for the health prediction model

In this paper, the developed health prediction model is constructed based on BRB-r, which is an expert system. Experts determine the initial health prediction model and provide all reference points, output belief degrees for the belief rules, attribute weights, and rule weights. Given the fuzziness and nondeterminacy of the expert knowledge, the initial model for health prediction cannot accurately predict the health status of a network in an actual working environment. Therefore, monitoring data are required to adjust the parameters of the initial health prediction model. In addition, the most significant advantage of the BRB-r model is its interpretability. Thus, some constraints should be added to the modeling process.

Based on the above analysis, we conclude that the optimization model for the developed health prediction model contains a single optimization target and is a constrained optimization model. The P-CMA-ES algorithm is an intelligent optimization algorithm that can address the gradient diffusion problem of the BRB-r model. Thus, in this paper, the optimization model for the health prediction model is proposed based on the P-CMA-ES algorithm.

The mean square error (MSE) represents the error between the actual health status and the estimated output of the constructed model. It is applied to express the accuracy of the model and can be calculated as follows^13^.

15 $$ MSE = \frac{1}{T}\sum\limits_{t = 1}^{T} {(output_{estimated} (t) - output_{actual} (t))^{2} } $$

where $T$ is the number of monitoring data; $output_{estimated} (t)$ and $output_{actual} (t)$ are the estimated model output and actual model output at time instant $t$, respectively.

To ensure the physical meaning of the health prediction model parameters, the following constraints should be provided.

16 $$ \begin{array}{*{20}c} {0 \le \theta_{k} \le 1,} & {k = 1,2, \ldots ,L} \\ \end{array} $$

17 $$ 0 \le \delta_{i} \le 1,\quad i = 1, \ldots ,t - 1 $$

18 $$ 0 \le \beta_{n,k} \le 1,\quad n = 1, \ldots ,N,\quad k = 1,2, \ldots L $$

19 $$ \sum\limits_{n = 1}^{N} {\beta_{n,k} } \le 1,\quad k = 1,2, \ldots ,L $$

The optimization model for the health prediction model can be profiled as

20 $$ \begin{aligned} & \min \;MSE(\theta_{k} ,\beta_{n,k} ,\delta_{i} ) \\ & {\text{s}}.{\text{t}}. \quad \begin{array}{*{20}c} {0 \le \theta_{k} \le 1,} & {k = 1,2, \ldots ,L} \\ \end{array} \\ & \quad \quad 0 \le \delta_{i} \le 1,\quad i = 1, \ldots ,t - 1 \\ & \quad \quad 0 \le \beta_{n,k} \le 1,\quad n = 1, \ldots ,N,\quad k = 1,2, \ldots L \\ \end{aligned} $$

In this paper, the estimated output of the health prediction model is obtained by Eq. (14), and the actual health status is provided by experts.

### Modeling process of the developed health prediction model

The modeling process of the proposed health prediction model includes two parts: training and testing. The detailed inference process consists of three steps.

- *Step 1* Gather the monitoring data and build the dataset. In the health status prediction model, the observation data are obtained by sensors, and key features should be selected as inputs for the BRB-r model.
- *Step 2* Calculate the reliabilities of the features using the proposed methods in "The average distance-based feature reliability calculation method" section and construct the initial health prediction model by using expert knowledge, as shown in Eq. (4). The output the belief degrees of belief rules, reference points, attribute weights, and rule weights are provided by experts according to the actual working environment of the sensor network.
- *Step 3* Use the optimized model to train the health prediction model. During the training process, the parameters in the health prediction model should satisfy the constraints in Subsection 3.3.
- *Step 4* Test the optimization model. After training the health prediction model, the optimized model can be tested on the testing data sets. The reliability of the features is the objective, and it is not affected by the testing data or the training data.

## Case study

In this section, to illustrate the applicability of the proposed health prediction model for a sensor network, a case study regarding a wireless sensor network (WSN) for oil storage tanks is provided.

### Problem formulation for predicting the health of the WSN

Oil storage tanks are used to store oil, and the number of such tanks is increasing. WSNs are used to monitor their safety and reliability. Therefore, the reliability of the WSN is the basis to determine the reliability of the oil storage tank. In this section, a health prediction model for the WSN is proposed based on the BRB-r developed in this paper.

The experiment was conducted based on an oil storage tank in Hainan, China. The oil storage tank was built at the seaside, and long working hours may cause the tank to leak, which makes the health prediction and maintenance of the tank necessary and essential. However, there are three problems in health prediction. First, the cost of experimentation is high, and due to the high reliability of the tank, the probability of failure is low. Thus, large amounts of observation data are not accessible. Second, WSNs for oil storage tanks are widely distributed, and many factors affect their working states, which makes it impossible to obtain accurate knowledge regarding their health statuses. Finally, a WSN relies on wireless transmission. The transmission process is interfered with by environmental factors, and there is noise in the observation information, which decreases the reliability of the information. Thus, a health prediction model that can address these three problems is required and can be constructed by the developed model.

### Construction of the health prediction model

In this experiment, the failure rate ($FR$) and available range ($AR$) were selected as two features of the WSN. Based on the network's observation information, four and five reference points were used, and the reference values were provided by experts, as shown in Tables 1 and 2, respectively. $H$, $SH$, $SL$, $M$ and $L$ represent high, slightly high, medium, slightly low, and low, respectively. The model complexity and prediction accuracy should be considered when calculating the number of reference points. Based on the form of the belief rules, as shown by Eq. (2), the belief rules in the health prediction model for the WSN of the oil storage tank can be formulated as follows:

21 $$ \begin{aligned} & R_{k} :{\text{If}}\;x_{AR} (t){\text{is }}A_{1}^{k} \wedge x_{FR} (t){\text{ is }}A_{2}^{k} {\text{, Then }}H(t + 1){\text{ is }}\left\{ {\left( {D_{1} ,\beta_{1,k} } \right),\left( {D_{2} ,\beta_{2,k} } \right),\left( {D_{3} ,\beta_{3,k} } \right)} \right\} \\ & \quad \;\;{\text{With}}\;{\text{rule}}\;{\text{weight }}\theta_{k} {\text{, characteristic}}\;{\text{weight }}\delta_{1} ,\delta_{2} ,\;{\text{characteristic}}\;{\text{reliability}}\;r_{1} ,r_{2} \\ \end{aligned} $$

**Table 1:** Reference points and values for $FR$.

Reference point	$H$	$SH$	$M$	$SL$	$L$
Reference value	0.0944	0.06	0.045	0.03	0.003

**Table 2:** Reference points and values for $AR$.

Reference point	$H$	$SH$	$M$	$L$
Reference value	65.63	31.24	9.38	3.12

**Figure 2:**
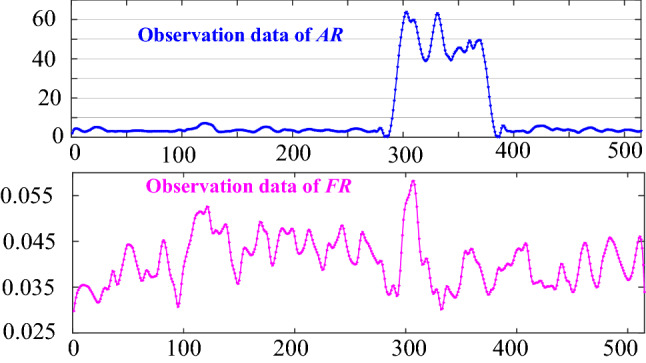
Observation data of two features.

As shown in this formula, the observation information regarding $FR$ and $AR$ at time instant $t$ is used as the input of the BRB-r model. In the experiment, three health grades were selected in the output of the belief rule as shown in Table 3, where $H$, $M$ and $L$ represent high, medium, and low, respectively. The output belief degree of the belief rule was provided by experts according to the working state of the WSN, and the initial health prediction model is shown in Table 4. In the initial health prediction model, the belief rules are considered crucial, and their rule weights are taken as 1. The observation data of the two features are shown in Fig. 2. The reliabilities of the monitoring data regarding $FR$ and $AR$ are calculated based on the proposed method, and the mean reliabilities are 0.3483 and 0.2517, respectively.

**Table 3:** Reference points for the output grades.

Reference point	$H$	$M$	$L$
Reference value	1	0.5	0

**Table 4:** Initial health prediction model for the WSN.

No	Rule weight	Feature		Output distribution	No	Rule weight	Feature		Output distribution
		$AR$	$FR$	$\{ H,\;M,\;L\}$			$AR$	$FR$	$\{ H,\;M,\;L\}$
1	1	$L$	$L$	(1 0 0)	11	1	$SH$	$L$	(0.2 0.8 0)
2	1	$L$	$SL$	(0.8 0.2 0)	12	1	$SH$	$SL$	(0 1 0)
3	1	$L$	$M$	(0.6 0.4 0)	13	1	$SH$	$M$	(0 0.8 0.2)
4	1	$L$	$SH$	(0.5 0.5 0)	14	1	$SH$	$SH$	(0 1 0)
5	1	$L$	$H$	(0.4 0.6 0)	15	1	$SH$	$H$	(0 0.8 0.2)
6	1	$M$	$L$	(0.35 0.75 0)	16	1	$H$	$L$	(0 0.9 0.1)
7	1	$M$	$SL$	(0.2 0.8 0)	17	1	$H$	$SL$	(0 0.8 0.2)
8	1	$M$	$M$	(0.1 0.9 0)	18	1	$H$	$M$	(0 0.5 0.5)
9	1	$M$	$SH$	(0 0.7 0.3)	19	1	$H$	$SH$	(0 0.2 0.8)
10	1	$M$	$H$	(0 0.5 0.5)	20	1	$H$	$H$	(0 0 1)

### Training and testing the health prediction model

In total, 515 sets of monitoring data were collected in this experiment. According to the number of optimization parameters in the health prediction model, 250 sets of monitoring data were randomly selected as the training data, and 265 sets of monitoring data were used as the testing data. In the optimization model constructed based on the P-CMA-ES algorithm, the output belief degrees of the belief rules, rule weights, and feature weights were selected as the optimization parameters. There were 82 optimization parameters in the health prediction model for the WSN.

**Figure 3:**
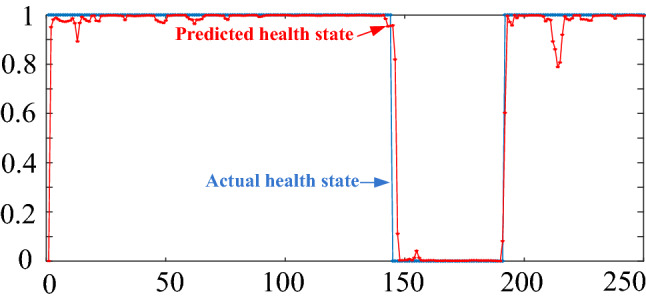
Predicted health status based on the proposed model.

The experiment was repeated 50 times. The minimum MSE was 0.0077. The variation in the MSEs was 1.5722E-07. The mean of the MSEs was 0.0084. This result proves that the developed BRB-r-based prediction model can accurately predict the health status of a WSN. The optimized health prediction model for the examined WSN is shown in Table 5. The optimized weights of $AR$ and $FR$ were 0.9998 and 0.1294, respectively. Figure 3 shows that the new BRB-r-based prediction method can accurately predict the health status of the WSN.

**Table 5:** Optimized health prediction model for WSN.

No	Rule weight	Feature		Output distribution
		$AR$	$FR$	$\{ H,\;M,\;L\}$
1	0.4857	$L$	$L$	(0.9248 0.0072 0.0680)
2	0.2223	$L$	$SL$	(0.9293 0.0571 0.0136)
3	0.0030	$L$	$M$	(0.3626 0.0091 0.6283)
4	0.0019	$L$	$SH$	(0.8781 0.1161 0.0057)
5	0.3637	$L$	$H$	(0.0000 0.1918 0.8085)
6	0.0000	$M$	$L$	(0.1931 0.8066 0.0003)
7	0.2912	$M$	$SL$	(0.9985 0.0025 0.0000)
8	0.9306	$M$	$M$	(0.9992 0.0011 0.0000)
9	0.6959	$M$	$SH$	(0.9963 0.0039 0.0000)
10	0.5965	$M$	$H$	(0.9952 0.0036 0.0012)
11	0.9973	$SH$	$L$	(0.0003 0.0014 0.9983)
12	0.0000	$SH$	$SL$	(0.0014 0.0016 0.9970)
13	0.3308	$SH$	$M$	(0.0053 0.0322 0.9625)
14	0.0137	$SH$	$SH$	(0.5073 0.4924 0.0003)
15	0.6357	$SH$	$H$	(0.1238 0.3275 0.5487)
16	0.0036	$H$	$L$	(0.0721 0.2685 0.6594)
17	0.8106	$H$	$SL$	(0.0016 0.0018 0.9966)
18	0.8338	$H$	$M$	(0.0000 0.0042 0.9964)
19	0.9966	$H$	$SH$	(0.0033 0.0012 0.9954)
20	0.0000	$H$	$H$	(0.0384 0.5554 0.4062)

## Conclusion

In practice, the observation information of a complex system is collected by using a sensor network, and this information provides a reference for decision making. This paper proposes a new health prediction model built on the belief rule base with attribute reliability (BRB-r), where an original calculation method for feature reliability is presented based on the average distance of the observation data.

The main innovations of this paper can be concluded as the following three points. First, a new health prediction model based on BRB-r is established to address the three problems in practical systems: the lack of observation data, high complexity of the network and environmental noise. Second, the sensor network is used to monitor the state of the system. When the system state changes, the observation data gathered by the network also change. The average distance of the observation data can reflect the degree of unreliability of the feature. Thus, according to the characteristics of the sensor network, a new feature reliability calculation method is developed based on the average distance of the observation data. Third, to overcome the fuzziness and nondeterminacy of expert knowledge, an optimization model is constructed based on the P-CMA-ES optimization algorithm.

This article assumes that the features of sensor networks are independent, and their correlations are not considered. In addition, interference factors in the real world may cause data loss. These issues pose challenges for future studies.
